# Clinical Significance of the Decreased Expression of hsa_circ_001242 in Oral Squamous Cell Carcinoma

**DOI:** 10.1155/2018/6514795

**Published:** 2018-07-04

**Authors:** Shuai Sun, Bowen Li, Yufan Wang, Xiang Li, Panpan Wang, Feng Wang, Wei Zhang, Hongyu Yang

**Affiliations:** ^1^Peking University Shenzhen Hospital Clinical College, Anhui Medical University, Hefei, Anhui, China; ^2^Department of Oral and Maxillofacial Surgery, Peking University Shenzhen Hospital, Shenzhen, Guangdong, China; ^3^Biomedical Research Institute, Shenzhen Peking University-The Hong Kong University of Science and Technology Medical Center, Shenzhen, China

## Abstract

**Background:**

Circular RNAs (circRNAs) are a type of covalently closed loop structure of endogenous RNAs. Recent studies have shown that circular RNAs may play an important role in human cancer. However, there is limited information on the function of circRNA in oral squamous cell carcinoma (OSCC).

**Methods:**

Hsa_circ_001242 expression levels in 40 paired OSCC tissues and four OSCC cell lines were selected using real-time quantitative reverse transcription polymerase chain reaction (qRT-PCR). A receiver operating characteristic (ROC) curve was used to evaluate the diagnostic value of hsa_circ_001242 in OSCC.

**Results:**

Hsa_circ_001242 was significantly downregulated in OSCC tissues compared to paired adjacent normal tissues (*P* < 0.001). Hsa_circ_001242 expression levels were significantly downregulated in four OSCC cell lines (SCC-9, SCC-15, SCC25, and CAL-27) than in human normal oral keratinocyte (HOK) cell lines. Moreover, the expression level of hsa_circ_001242 was negatively correlated with tumor size and T stage (*P* < 0.05). The area under the ROC curve was 0.784.

**Conclusion:**

This study showed that hsa_circ_001242 was significantly downregulated in OSCC and may act as a potential novel biomarker for the diagnosis and treatment of OSCC.

## 1. Introduction

Oral squamous cell carcinoma (OSCC) is the third most common cancer in developing countries and ranks sixth among systemic cancers worldwide [1, 2]. Although there have been some improvements in the diagnosis and clinical treatment of OSCC, the 5-year overall survival rate of OSCC patients has not improved and remains <50% over the last three decades [3, 4]. In addition, many patients have a poor response to therapy and high recurrence rates [5]. Therefore, it is important to determine effective molecular diagnostic markers and therapeutic targets of OSCC.

Circular RNAs (circRNAs) are a type of covalently closed loop structure of endogenous RNAs, which are characterized by linking the 3′ and 5′ ends generated by backsplicing. Unlike linear RNAs, circRNAs require high conservation, have high stability and tissue specificity, and are not easily degraded by enonuclease [6, 7]. In the 1970s, circRNAs were first discovered in Sendai viruses and then were clearly observed in eukaryotes [8, 9]. CircRNAs were initially deemed to the products of aberrant RNA splicing, without attracting attention [10]. With the development of RNA high-throughput sequencing technology and bioinformatics, thousands of circRNAs have been discovered in the human genome [11]. Numerous researches have shown that circRNAs can be used as new biomarkers for clinical diagnosis and treatment of cancer [12–14]. However, there is little known about the role of circRNA in OSCC.

**Figure 1:**
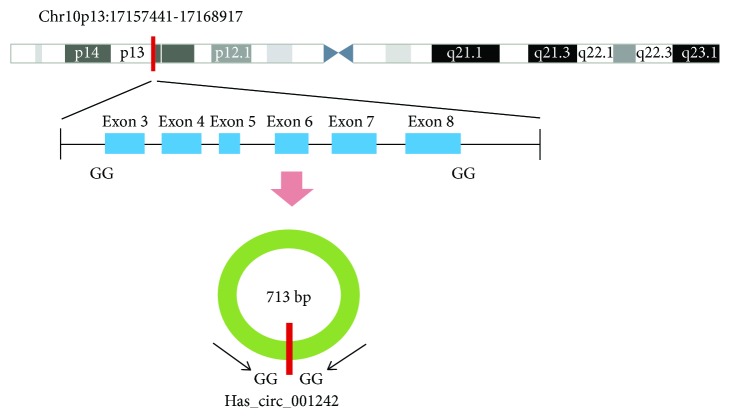
Hsa_circ_001242 is encoded from chromosomal region 10p13. Six exons of them form hsa_circ_001242 from exon 3 to exon 8.

Searching OSCC associated circRNAs from circBase [15]. We first confirmed that hsa_circ_001242, which is located at chr10: 17157441–17168917 (Figure 1), was significantly downregulated in OSCC cell lines and OSCC tissues. Hsa_circ_001242, with a spliced sequence length of 713 bp, associated gene symbol is TRDMT1 (tRNA aspartic acid methyltransferase 1) and composed of six exons from exon 3 to exon 8. Its expression levels in tissues at various stages of OSCC were then explored, and the potential relationship between hsa_circ_001242 expression levels and patients' clinicopathological factors were investigated. A receiver operating characteristic (ROC) curve was constructed for evaluating the diagnostic value of hsa_circ_001242. Our data indicate that hsa_circ_001242 may be a potential biomarker for the diagnosis of OSCC.

## 2. Materials and Methods

### 2.1. Patients and Specimens

Forty oral cancer tissue samples and paired adjacent normal tissues were collected from the Department of Oral and Maxillofacial Surgery, Shenzhen Hospital, Peking University (Shenzhen, China), from December 2016 to May 2017. All samples were verified by histopathology. No patient previously underwent OSCC surgery, chemotherapy, or radiotherapy. Tissue samples were stored at −80°C before use. This study was approved by Ethics Committee of Peking University Health Science Center (IRB00001053-08043).

### 2.2. RNA Extraction

Total RNA was extracted from OSCC tissues and paired adjacent normal tissues using the TRIzol reagent (Invitrogen, Carlsbad, CA, USA) according to the manufacturer's protocol. Total RNA from each specimen was quantified, and quality assurance was conducted using a NanoDrop ND­2000 spectrophotometer (NanoDrop, Wilmington, DE, USA). Reaction mixture (20 *μ*l) containing 1 *μ*g total RNA was reverse transcribed to cDNA using PrimeScript RT-polymerase (Takara, Dalian, China).

The RNA sample was dissolved in RNase-free water. The yield and purity were measured using a NanoDrop 2000 instrument (Thermo Fisher Scientific, Waltham, MA, USA). The integrity of the RNA was determined using 1% formaldehyde denaturing gel electrophoresis. A PrimeScript RT Reagent Kit (Takara Bio, Nojihigashi, Kusatsu, Japan) was used for the production of complementary DNA (cDNA) by reverse transcription, according to the manufacturer's instructions. qRT-PCR was performed using SYBR-Green Premix Ex Taq (Takara Bio, Nojihigashi, Kusatsu, Japan) and was monitored using the ABI PRISM 7500 Sequence Detection System (Applied Biosystems, Life Technologies, Waltham, MA, USA). The relative expression levels of circRNAs were determined by qRT-PCR. The sequences of the primers used in the qRT-PCR assay are shown in Supplementary Table 1.

The sequences of the hsa_circ_001242 primers were as follow: 5′-GCCCACTTGTAGAAGGTCCG-3′ (forward primer) and reverse 5′-CTGGCAGGGAGGGCTCATTA-3′ (reverse primer). The primer sequences for *β*-actin were 5′-AAACTGGAACGTTGAGAGTG-3′ (forward primer) and 5′-AGTGGTCTGGCTTTTAGGT-3′ (reverse primer). The reaction conditions were as follows: 95°C at 5 min for a preincubation and 40 cycles of 95°C for 5 s, annealing temperature of 60°C for primer pairs for 30 s, and 72°C for 20 s. RNA levels were normalized using *β*-actin as the internal control.

### 2.3. Cell Culture

The human OSCC cell lines, SCC9, SCC15, SCC25, and CAL27, were obtained from the College of Stomatology, Wuhan University (Wuhan, China). Human oral keratinocytes (HOK) cells were obtained from the cell bank of the Chinese Academy of Sciences (Shanghai, China). SCC9 cells were cultured in DMED/F12 medium supplemented with 1% penicillin/streptomycin. SCC15, SCC25, CAL27, and HOK cells were cultured in Dulbecco's Modified Eagle Medium (DMED, GIBCO, China) supplemented with 1% penicillin/streptomycin (Life Technologies Inc., USA). All cells were cultured at 37°C under 5% CO_2_.

### 2.4. Statistical Analysis

GraphPad Prism 5.0 Software (GraphPad Software, La Jolla, CA, USA) was used to analyze the obtained data. Results of hsa_circ_001242 expression for OSCC tissues and paired adjacent normal tissues or between OSCC cell lines and HOK cell lines were compared using a paired *t*-test. A nonpaired *t*-test was used to analyze the relationship between hsa_circ_001242 expression level and clinicopathological factors in OSCC patients. The ROC curve was constructed to evaluate the diagnostic values. ^∗^ *P* < 0.05, ^∗∗^ *P* < 0.01, and ^∗∗∗^ *P* < 0.001 were considered statistically significant.

## 3. Results

### 3.1. Expression of hsa_circ_001242 in OSCC Tissues

**Figure 2:**
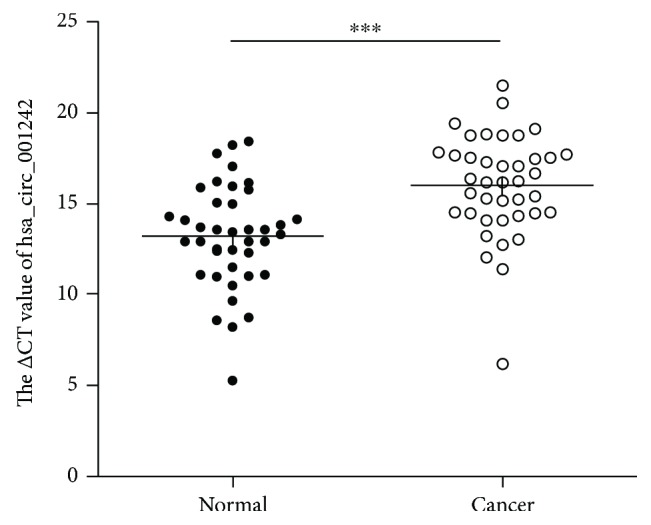
Hsa_circ_001242 expression levels in 40 pairs of OSCC tissues compared to that in paired adjacent normal tissues. Higher △Ct value indicates lower expression. Data are expressed as mean ± SD; ^∗∗∗^ *P* < 0.001.

In this study, we first observed that the expression levels of hsa_circ_001242 were evidently downregulated in 40 OSCC tissue samples compared to that in paired adjacent normal tissues by qRT-PCR (*n* = 40, *P* < 0.001) (Figure 2).

### 3.2. Hsa_circ_001242 Expression Levels in OSCC Cell Lines

**Figure 3:**
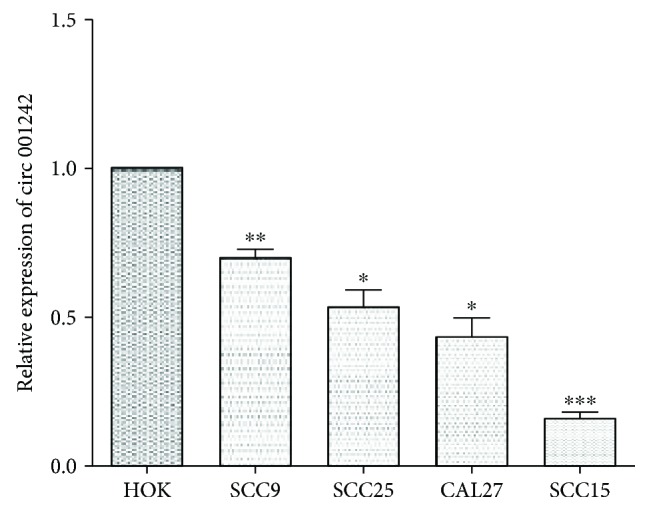
Hsa_circ_001242 expression in different OSCC cell lines. Hsa_circ_001242 expression levels in four OSCC cell lines (SCC-9, SCC-15, SCC-25, and CAL-27), and normal human oral keratinocyte cell lines were assessed by qRT-PCR. Data are presented as mean ± SD; ^∗^ *P* < 0.05, ^∗∗^ *P* < 0.01, and ^∗∗∗^ *P* < 0.001.

Furthermore, we explored the expression levels of *hsa_circ_001242* in the HOK cell lines and four human OSCC cell lines, SCC-9, SCC-15, SCC-25, and CAL27. Expression levels in the OSCC cell lines were significantly lower than those in the HOK cell lines (Figure 3).

### 3.3. Relationship between hsa_circ_001242 Levels and Clinicopathological Factors

Clinicopathological analysis revealed that hsa_circ_001242 expression level was significantly associated with clinicopathological factors of OSCC patients. As presented in Table 1, hsa_circ_001242 expression levels were negatively related to tumor size (*P* = 0.0125) and T stage (*P* = 0.0434) but were not associated with other clinicopathological features, such as age, gender, tumor differentiation, TNM stage, or lymphatic metastasis (*P* > 0.05).

### 3.4. The Diagnostic Value of hsa_circ_001242 in OSCC

**Figure 4:**
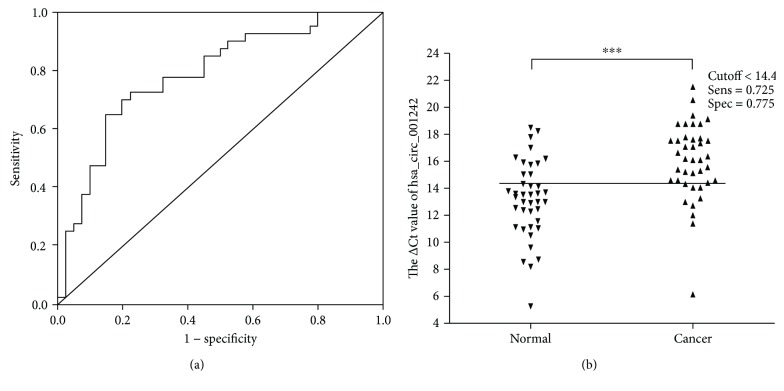
The diagnostic value of hsa_circ_001242 in OSCC. (a) The area under the ROC curve (AUC) was 0.784 (95% CI = 0.683–0.885, *P* < 0.001), (b) and the cutoff of hsa_circ_001242 was 14.4. The sensitivity and specificity were 0.725 and 0.775, respectively. Data are presented as mean ± SD; ^∗∗∗^ *P* < 0.001.

To estimate the diagnostic value of hsa_circ_001242 in OSCC, an ROC curve was constructed for differentiating OSCC tissues from paired adjacent normal tissues. The area under the ROC curve (AUC) was 0.784 (95% confidence interval (CI) = 0.717–0.867; *P* < 0.001; Figure 4(a)). The cutoff value was 14.4 (Figure 4(b)), and the corresponding sensitivity and specificity of hsa_circ_001242 were 72.5% and 77.5%, respectively. When the area under the curve is larger, the diagnostic value of the variable is higher. Values below the cutoff value are negative, while those exceeding the cutoff value are positive. Therefore, hsa_circ_001242 may act as a potential biomarker for the diagnosis of OSCC.

## 4. Discussion

CircRNAs are a class of noncoding RNAs (ncRNAs) that have been neglected as transcriptional noise in eukaryotes for the past 30 years [10, 16, 17]. Recent studies have found that some circRNAs serve as an important regulatory role and do not splice noise as previously thought [18]. Compelling evidence has manifested that circRNA can regulate gene expression through multiple mechanisms. CircRNAs act as a sponge of miRNA molecules and competitively bind miRNAs to regulate gene expression [19, 20]. Exon-intron circRNAs (EIciRNAs) can regulate transcription by RNA-RNA interactions in the nucleus [21, 22]. Some reports showed that circRNAs have the function of being translated into proteins [23, 24]. These studies indicate that the circular RNA plays an important role in transcriptional and posttranscriptional levels and can serve as an ideal marker for disease diagnosis. Previous studies have suggested that circRNAs may play an important role in the development of cancer and act as potential biomarkers for cancer [25, 26]. For example, Li Wan et al. [27] have revealed that circ-ITCH is overexpressed in lung cancer tissues and inhibits the Wnt/*β*-catenin signaling pathway by acting as a sponge for miR-7 and miR-214. Zhu et al. [28] have reported that circ-BANP expression in colorectal cancerous tissues is significantly upregulated than those in adjacent normal tissues. Huang et al. [29] have observed that hsa_circ_0000745 is downregulated in gastric cancer tissues as well as in the plasma samples from patients with gastric cancer. Their study revealed that the circular RNA hsa_circ_0000745 may serve as a diagnostic marker for gastric cancer. Yao et al. [30] have confirmed that circZKSCAN1 is downregulated in hepatocellular carcinoma and may act as a diagnostic biomarker. Thus, we believe that circRNAs may serve as novel molecular markers for cancer.

In this study, we first observed that hsa_circ_001242 was downregulated in both OSCC tissues and OSCC cell lines. Furthermore, the study on the clinical features and the expression of hsa_circ_001242 indicated that hsa_circ_001242 expression level was negatively relevant to tumor size and T stage (Table 2). These data suggest that circRNAs may play a role in carcinogenesis and progress of OSCC. The ROC curve was constructed for differentiating OSCC tissues from the controls. Our results demonstrated that AUC was 0.784. The sensitivity and specificity of hsa_circ_001242 were 0.725 and 0.775, respectively. Furthermore, we observed that the cutoff value of hsa_circ_001242 was 14.4. To further understand the biological role of circRNAs in OSCC, both in vitro and in vivo assays should be performed in the future after successfully engineering of circRNAs.

In conclusion, our study manifested that hsa_circ_001242 was significantly downregulated in OSCC tissues and OSCC cell lines. In addition, the expression of hsa_circ_001242 expression is negatively correlated with tumor size and T stage. Thus, hsa_circ_001242 may serve as a potential diagnostic biomarker for OSCC.

## Figures and Tables

**Table 1:** Primer sequences.

Primer set	Forward primer	Reverse primer
hsa_circ_001242	GCCCACTTGTAGAAGGTCCG	CTGGCAGGGAGGGCTCATTA
*β*-Actin	AAACTGGAACGTTGAGAGTG	AGTGGTCTGGCTTTTAGGT

**Table 2:** Correlation between clinicopathological factors and hsa_circ_001242 expression levels (ΔCt) in oral squamous cell carcinoma patients.

Characteristics	Number of patients	Mean ± SD	*P* value
Age (year)			
≥60	14	16.88 ± 0.644	0.145
<60	26	15.52 ± 0.572	
Gender			
Male	28	16.05 ± 0.537	0.871
Female	12	15.89 ± 0.813	
Tumor size(cm)			
≥5	6	13.43 ± 0.543	0.0125^∗^
<5	34	16.45 ± 0.408	
Differentiation			
Well and moderate	34	16.23 ± 0.424	0.221
Poor	6	14.70 ± 0.747	
T stage			
T1–2	29	16.58 ± 0.45	0.0317^∗^
T3–4	11	14.47 ± 0.99	
TNM			
I and II	19	16.76 ± 0.61	0.103
III and IV	21	15.31 ± 0.61	
Lymphatic metastasis			
N0	24	16.50 ± 0.516	0.170
N1–3	16	15.25 ± 0.773	

^∗^ indicated statistical significance.

## Data Availability

The data used to support the findings of this study are available from the corresponding author upon request.

## Supplementary Materials

Supplementary Materials Supplementary Information Table 1. Detailed information on clinical and oral squamous cell carcinoma characteristics of patients and the information of metastasis.
