# Corrigendum to “Clinical Significance of the Decreased Expression of hsa_circ_001242 in Oral Squamous Cell Carcinoma”

**DOI:** 10.1155/2019/2434063

**Published:** 2019-05-09

**Authors:** Shuai Sun, Bowen Li, Yufan Wang, Xiang Li, Panpan Wang, Feng Wang, Wei Zhang, Hongyu Yang

**Affiliations:** ^1^Peking University Shenzhen Hospital Clinical College, Anhui Medical University, Hefei, Anhui, China; ^2^Department of Oral and Maxillofacial Surgery, Peking University Shenzhen Hospital, Shenzhen, Guangdong, China; ^3^Biomedical Research Institute, Shenzhen Peking University-The Hong Kong University of Science and Technology Medical Center, Shenzhen, China

In the article titled “Clinical Significance of the Decreased Expression of hsa_circ_001242 in Oral Squamous Cell Carcinoma” [1], the tables were incorrectly cited. The tables' correct in-text citations are as follows:

1. In Section 2.1 titled “Patients and Specimens,” the citation of the Supplementary Table 1 should be added as follows: “Forty oral cancer tissue samples and paired adjacent normal tissues were collected from the Department of Oral and Maxillofacial Surgery, Shenzhen Hospital, Peking University (Shenzhen, China), from December 2016 to May 2017 (Supplementary Table 1)”
2. In Section 2.2 titled “RNA Extraction,” “The sequences of the primers used in the qRT-PCR assay are shown in Supplementary Table 1” should be corrected to “The sequences of the primers used in the qRT-PCR assay are shown in Table 1”
3. In Section 3.3 titled “Relationship between hsa_circ_001242 Levels and Clinicopathological Factors,” “As presented in Table 1” should be corrected to “As presented in Table 2.” Also, “T stage (*P* = 0.0434)” should be corrected to “T stage (*P* = 0.0317)”
