# Supplementary material for: Clinical Significance of the Decreased Expression of hsa_circ_001242 in Oral Squamous Cell Carcinoma
Source: Dis Markers. 2018 Jul 4;2018:6514795. doi: 10.1155/2018/6514795 (PMC6057325; doi:10.1155/2018/6514795)
Supplement: Supplementary Materials — Supplementary Information Table 1. Detailed information on clinical and oral squamous cell carcinoma characteristics of patients and the information of metastasis. [file 6514795.f1.docx]

**Supplementary table 1. Detailed information of clinical and Oral Squamous Cell Carcinoma characteristics of patients and the information of metastasis.**

|  | **Gende** | **Age** | **Location** | **Tumor size(cm)** | **TNM** | **Lymph node metastasis** | **Distant metastasis** | **Clinical stage** |
| --- | --- | --- | --- | --- | --- | --- | --- | --- |
| 1 | male | 65 | tongue | <5 | T2N0M0 | none | none | Ⅱ |
| 2 | female | 58 | gingive | ≥5 | T4N0M0 | none | none | Ⅳ |
| 3 | male | 57 | tongue | <5 | T2N0M0 | none | none | Ⅱ |
| 4 | female | 76 | cheek | ≥5 | T3N0M0 | none | none | Ⅲ |
| 5 | male | 53 | oropharynx | ≥5 | T4N2M0 | homolateral, multiple ,＜6cm | none | Ⅳ |
| 6 | female | 60 | cheek | ≥5 | T4N2M0 | homolateral, multiple ,＜6cm | none | Ⅳ |
| 7 | male | 58 | tongue | <5 | T3N1M0 | homolateral, single,2cm | none | Ⅲ |
| 8 | male | 48 | tongue | <5 | T2N0M0 | none | none | Ⅱ |
| 9 | male | 53 | cheek | <5 | T4aN1M0 | homolateral, multiple ,＜6cm | none | Ⅳ |
| 10 | female | 69 | tongue | <5 | T2N2M0 | homolateral, multiple ,＜6cm | none | Ⅳ |
| 11 | female | 68 | gingive | <5 | T4N0M0 | none | none | Ⅳ |
| 12 | male | 32 | tongue | <5 | T2N0M0 | none | none | Ⅱ |
| 13 | male | 33 | tongue | <5 | T1N1M0 | homolateral, single,2cm | none | Ⅲ |
| 14 | male | 39 | gingive | <5 | T2N1M0 | homolateral, single,2cm | none | Ⅲ |
| 15 | male | 60 | tongue | <5 | T1N0M0 | none | none | Ⅰ |
| 16 | male | 51 | mouth floor | <5 | T2N0M0 | none | none | Ⅱ |
| 17 | male | 50 | cheek | <5 | T1N0M0 | none | none | Ⅰ |
| 18 | male | 60 | tongue | ≥5 | T3N0M0 | none | none | Ⅲ |
| 19 | female | 66 | gingive | <5 | T4N0M0 | none | none | Ⅳ |
| 20 | male | 32 | tongue | <5 | T2N1M0 | homolateral, single,2cm | none | Ⅲ |
| 21 | male | 53 | tongue | <5 | T4aN2M0 | homolateral, single,2cm | none | Ⅳ |
| 22 | male | 54 | mouth floor | <5 | T2N0M0 | none | none | Ⅱ |
| 23 | male | 53 | tongue | <5 | T2N2M0 | homolateral,  multiple ,＜6cm | none | Ⅳ |
| 24 | female | 55 | cheek | <5 | T2N0M0 | none | none | Ⅱ |
| 25 | female | 31 | tongue | <5 | T2N0M0 | none | none | Ⅱ |
| 26 | male | 40 | tongue | <5 | T2N0M0 | none | none | Ⅱ |
| 27 | male | 40 | tongue | <5 | T1N1M0 | homolateral, single,2cm | none | Ⅲ |
| 28 | male | 29 | tongue | <5 | T2N0M0 | none | none | Ⅱ |
| 29 | male | 78 | oropharynx | ≥5 | T4aN2M0 | homolateral, single,2cm | none | Ⅳ |
| 30 | male | 49 | tongue | <5 | T2N1M0 | homolateral, single,2cm | none | Ⅲ |
| 31 | male | 60 | mouth floor | <5 | T2N1M0 | homolateral, single,2cm | none | Ⅲ |
| 32 | female | 73 | tongue | <5 | T2N1M0 | homolateral, single,2cm | none | Ⅲ |
| 33 | male | 45 | tongue | <5 | T2N0M0 | none | none | Ⅱ |
| 34 | male | 50 | tongue | <5 | T2N0M0 | none | none | Ⅱ |
| 35 | female | 64 | cheek | <5 | T2N0M0 | none | none | Ⅱ |
| 36 | male | 36 | tongue | <5 | T1N0M0 | none | none | Ⅰ |
| 37 | female | 60 | cheek | <5 | T1N0M0 | none | none | Ⅰ |
| 38 | male | 63 | tongue | <5 | T1N0M0 | none | none | Ⅰ |
| 39 | male | 59 | mouth floor | <5 | T2N2bM0 | bilateral,  multiple,＜6cm | none | Ⅳ |
| 40 | female | 47 | tongue | <5 | T2N0M0 | none | none | Ⅱ |
